# Feasibility of Transport of 26 Biologically Active Ultrashort Peptides via LAT and PEPT Family Transporters

**DOI:** 10.3390/biom13030552

**Published:** 2023-03-17

**Authors:** Vladimir Khatskelevich Khavinson, Natalia Sergeevna Linkova, Andrey Ivanovich Rudskoy, Michael Gennadievich Petukhov

**Affiliations:** 1 Department of Biogerontology, Saint Petersburg Institute of Bioregulation and Gerontology, 197110 Saint Petersburg, Russia; 2 Group of Peptide Regulation of Aging, Pavlov Institute of Physiology of Russian Academy of Sciences, 199034 Saint Petersburg, Russia; 3 The Department of Therapy, Geriatrics and Anti-Age Medicine, Academy of Postgraduate Education under of FSBU FSCC of FMBA of Russia, 125371 Moscow, Russia; 4 Group of Biophysics, Higher Engineering and Technical School, Peter the Great St., Petersburg Polytechnic University, 195251 Saint Petersburg, Russia; 5 Petersburg Nuclear Physics Institute Named after B.P. Konstantinov, NRC “Kurchatov Institute”, 188300 Gatchina, Russia

**Keywords:** ultrashort peptides, LAT, PEPT, peptide transport into the cell, molecular modeling

## Abstract

The aim of this work is to verify the possibility of transport of 26 biologically active ultrashort peptides (USPs) into cells via LAT and PEPT family transporters. Molecular modeling and computer-assisted docking of peptide ligands revealed that the size and structure of ligand-binding sites of the amino acid transporters LAT1, LAT2, and of the peptide transporter PEPT1 are sufficient for the transport of the 26 biologically active di-, tri-, and tetra-peptides. Comparative analysis of the binding of all possible di- and tri-peptides (8400 compounds) at the binding sites of the LAT and PEPT family transporters has been carried out. The 26 biologically active USPs systematically showed higher binding scores to LAT1, LAT2, and PEPT1, as compared with di- and tri-peptides, for which no biological activity has been established. This indicates an important possible role which LAT and PEPT family transporters may play in a variety of biological activities of the 26 biologically active peptides under investigation in this study. Most of the 26 studied USPs were found to bind to the LAT1, LAT2, and PEPT1 transporters more efficiently than the known substrates or inhibitors of these transporters. Peptides ED, DS, DR, EDR, EDG, AEDR, AEDL, KEDP, and KEDG, and peptoids DS7 and KE17 with negatively charged Asp^−^ or Glu^−^ amino acid residues at the N-terminus and neutral or positively charged residues at the C-terminus of the peptide are found to be the most effective ligands of the transporters under investigation. It can be assumed that the antitumor effect of the KE, EW, EDG, and AEDG peptides could be associated with their ability to inhibit the LAT1, LAT2, and PEPT1 amino acid transporters. The data obtained lead to new prospects for further study of the mechanisms of transport of USP-based drugs into the cell and design of new antitumor drugs.

## 1. Introduction

Ultrashort peptides (USPs), consisting of 2–8 amino acid residues, regulate the functions of the endocrine, nervous, and immune systems, and are also involved in cell proliferation, differentiation, and apoptosis [1]. Therefore, the study on the mechanisms of USPs transport into cells with the participation of PEPT peptide transporters and LAT amino acid transporters is an urgent task for biophysics and molecular medicine.

The eukaryotic cell membrane is a barrier to amino acids, USPs, and other hydrophilic or charged substances. To maintain cell homeostasis over the course of evolution, a number of specialized proteins have been developed—membrane carriers of various biologically active substances. The ability of some positively charged short peptides to penetrate cells was first discovered in human immunodeficiency virus research. These peptides were called “cell-penetrating peptides”. They are used to transport various drugs into cells [2].

Fluorescent and electron microscopy, as well as modern methods of molecular modeling, revealed the passive transport of positively charged peptides in HeLa cells [3].

USPs are assumed to enter the cell with the participation of proton-dependent oligopeptide transporters (POT) [4]. The transport of USPs with the length of 2–3 amino acid residues can also be carried out with the participation of L-type amino acid transporters (LAT1 and LAT2). These amino acid transporters and USPs are essential for the nutrient supply of the cell, recycling of neurotransmitters, maintenance of redox balance, and intracellular signaling [4].

LATs are heterodimeric amino acid transporters. LAT1 predominantly transports neutral amino acids such as leucine, tryptophan, tyrosine, and phenylalanine, as well as some other biologically active substances, including L-DOPA, baclofen, anticonvulsants gabapentin, pregabalin, thyroid hormones T3, T4, and S-nitrosothiols, and the chemotherapy drug melphalan [5,6,7,8], using antiport mechanisms [9,10]. Histidine and tyrosine are transported by LAT1 bidirectionally, while other amino acids are predominantly transported into the cell [11]. Tyrosine derivative compound JPH203 (KYT-0353) is an effective selective inhibitor of LAT1. LAT1 blocking is of great therapeutic importance in conditions of increased metabolic activity and proliferation of cancer cells [12].

LAT2 has been found in almost all organs and tissues and possesses broad specificity for neutral amino acids, thyroid hormones, and amino acid-like xenobiotics [13] using an antiport mechanism. The LAT1 and LAT2 transporters are similar in structure and specificity of known substrates and have a fairly high level of amino acid sequence identity (~55%). Similar to LAT1, LAT2 has 12 transmembrane domains with N- and C-termini located intracellularly and forms a covalent heterodimer through a disulfide bridge with an accompanying protein (4F2 heavy chain), which is necessary for its localization in the plasma membrane, but not involved in the transport [14].

LAT2 is characterized by broad substrate specificity to small and large neutral (zwitterionic) amino acids (Tyr, Phe, Trp, Thr, Asn, Ile, Cys, Ser, Leu, Val, Gln, His, Ala, Met, Gln) [15], thyroid hormones (T3, 3,3′-diiodothyronine) [11,16], L-DOPA [9,10,17,18], and S-nitrosocysteine [6], as well as some exogenous amino acid-like xenobiotics. BCH (2-Aminobicyclo-(2,2,1)-heptane-2-carboxylic acid) and GPNA (l-γ-Glutamyl-p-nitroanilide) inhibit both LAT1 and LAT2 [19]. It is known that LAT1 is overexpressed in many types of cancer cells and contributes to tumor growth via the mTOR pathway. However, no similar universal role for LAT2 has been confirmed [20].

Di- and tri-peptides, as well as various peptide-like compounds, are transported into the epithelial cells of the intestine and kidneys via POT-type transporters, which include PEPT1 and PEPT2. In contrast, the level of amino acid sequence identity between the LAT and PEPT transporter families is quite low (~14–15%), which most likely means that they are not evolutionarily related. All the POT family members are similar in structure and contain 12 transmembrane domains with N- and C-termini facing the cell cytoplasm. PEPT1 and PEPT2 have high interspecific amino acid homology among mammals (~80%). However, the amino acid sequence homology between human PEPT1 and PEPT2 is relatively low (~50%) [21].

PEPT1 and PEPT2 possess high overlapping substrate specificity and are capable of transporting dipeptides and tripeptides regardless of the difference in their amino acid sequences [5,13,21,22]. The most pronounced PEPT1 expression is observed in the small intestine and kidneys. PEPT2 is predominantly expressed in the kidneys. Both transporters are localized in the brush border of epithelial cell membranes. PEPT1 is a low-affinity di- and tri-peptide transporter, while PEPT2 is a high-affinity transporter [21]. The PDB database contains spatial structures of LAT1, LAT2, PEPT1, and PEPT2 transporters in free form and in complex with peptides in various conformations of binding centers, characterizing the phases of substrate transfer through the cell membrane.

The aim of this work is to verify a possibility of transport of 26 biologically active USPs (AE, KE, KE-17, ED, DR, DA, DE, DG, DS, DS-7, EW, DW, DL, KED, EDG, EDR, EDL, AED, EDP, KEDP, AEDG, AEDR, AEDL, AEDP, KEDW-NH_2_, KEDA, KEDG) into cells via LAT and PEPT family transporters.

## 2. Materials and Methods

### 2.1. Molecular Modeling and Preparation of Receptors and Ligands

The study was carried out using computational methods of molecular modeling and ligand docking on a multiprocessor supercomputer of the Petersburg Nuclear Physics Institute Named after B.P. Konstantinov, NRC “Kurchatov Institute” (Gatchina, Russia). Regularized (full-atomic, having standard geometric characteristics and energetically minimized conformations at given pH values without omissions of structural elements) models of the LAT1, LAT2, PEPT1, and PEPT2 transporters’ spatial structures with different conformations of the ligand-binding sites were built on the basis of the structures available in the PDB database (PDB ID: 6IRS, 6IRT, 7DSK, 7DSL, 7DSN, 7DSQ, 7B00, 7CMH, 7CMI, 7PMW, 7PMX, 7PN1, and 7PMY) by means of the ICM-Pro (Molsoft LLC, USA) standard protocols and tools [23]. The standard torsion angles of the side chains of amino acid residues (χ_i_) varied during regularization and relaxation of the spatial structure of the protein under investigation and ligand docking in its active site. The standard energy parameters of the ECEPP/3 forcefield of the ICM-Pro software package for the Van der Waals, electrostatic, torsion, energy interactions, hydrogen bonds, and hydration were used in the calculations. The spatial structure library of the 26 studied USPs (AE, KE, KE-17, ED, DR, DA, DE, DG, DS, DS-7, EW, DW, DL, KED, EDG, EDR, EDL, AED, EDP, KEDP, AEDG, AEDR, AEDL, AEDP, KEDW-NH_2_, KEDA, KEDG) with free N- and C-termini was developed by means of the molecular editor integrated into the ICM-Pro software package. The spatial chemical structures of these compounds are presented in Table 1. In addition, libraries of spatial structures for all possible di- and tri-peptides including mirrored sequences (8400 compounds in total) were created for the comparative analysis of the USPs’ binding characteristics to LAT1, LAT2, PEPT1, and PEPT2 transporters.

### 2.2. Virtual Screening of Ligands

Ligand docking at the binding sites of LAT1, LAT2, PEPT1, and PEPT2 transporters with different active site conformations was performed by ICM-Dock method in the ICMFF forcefield using standard ICM protocols for flexible ligand docking implemented in the DockScan utility of the ICM-Pro software package. The ICM-Score function is the main indicator of quality and potential stability of the ligand-protein complexes under investigation. It is a partial analogue of the free binding energy and is measured in kcal/mol.

Over the course of the docking algorithm, a global optimization of the flexible ligand in the field of the active site of the receptor protein was carried out. In the first stage, the receptor potential map was calculated with respect to hydrogen bonds, Van der Waals, hydrophobic, and electrostatic interactions. After that, a conformational analysis of the ligand outside the receptor was performed to predict the initial conformations. The Monte Carlo method was used to change the internal set of variable bond angles of the ligand in the receptor field and to search for the local minimum with further minimization of the energy gradient. At the final stage, the energy function was calculated, on the basis of which a decision was made whether to reject or retain the obtained ligand conformation. The interaction energy between the ligand and the receptor was calculated as follows:

*ICM-Score* = ∆*E_vw_* + ∆*E_en_* + *α*_1_∆*E_at_* +*α*_2_∆*E_hb_* + *α*_3_∆*E_se_* + *α*_4_∆*E_el_* + *α*_5_∆*E_so_*

where: ∆*E_vw_*—the change in the Van der Waals interaction energy when a ligand is bound at the active site of a protein,

∆*E_en_*—the entropy contribution to the free binding energy associated with the loss of conformational freedom of the ligand and amino acid side chains at the active site of the protein resulting from the ligand binding to the receptor ($\frac{kT}{2}$ × the number of mobile torsion angles of the ligand),

*N_at_*—the number of ligand atoms,

∆*E_hb_*—the change in the hydrogen bond energy,

∆*E_se_*—the change in the electrostatic energy of hydration,

∆*E_el_*—the change in the energy of electrostatic interactions,

∆*E_so_*—the change in the hydrophobic interaction energy,

*α_i_*—empirically selected weight coefficients for the contributions of all of the above physical interactions to the free energy of ligand binding to the receptor protein.

As a rule, the lower the ICM-Score value, the better the binding energy for small drug-like ligands at the active sites of water-soluble proteins. It was previously shown that the ICM-Score values of drug-like ligands, capable of binding at the active sites of water-soluble proteins with binding constants at the level of ~10 µM, are ~−30 kcal/mol [24].

The search for the optimal low-energy conformations of the 26 USPs in the cavity of the ligand-binding site of LAT1, LAT2, PEPT1, and PEPT2 transporters was carried out using Monte Carlo methods with the maximum thoroughness criteria corresponding to the total number of moving angles for each ligand (thoroughness = 70). The angles were chosen to provide ≥90% reproducibility of the docking results [25].

The USP-binding sites were defined as a region within the cavity of the binding sites of the transporters under investigation, obtained from the spatial structures of their complexes with various substrates and inhibitors, presented in the PDB database. The cavities of the transporter-binding sites have hydrophobic, positively, and negatively charged regions, which provide a fairly large number of possibilities for the USPs’ binding. The lowest-energy USPs conformations in the cavity of the ligand-binding site of LAT1, LAT2, PEPT1, and PEPT2 transporters were selected for the further search of compounds that can participate in the transport through the cell membrane or block the transport mechanism of other ligands. These transporters were selected based on the comparison of the calculated ICM-Score values for the 26 USPs under investigation with the data obtained for known ligands and inhibitors of LAT1, LAT2, PEPT1, and PEPT2, as well as a comparative analysis of the obtained results with the results of docking of all possible di- and tri-peptides into the active sites of these proteins.

## 3. Results

### 3.1. Spatial Structures of UPS Complexes with LAT1, LAT2, PEPT1, and PEPT2 Transporters

The amino acid and peptide transporters under investigation have different amino acid sequences. However, their functional activity is similar. All these transporters have two major domains, one of which is embedded into the cell membrane in such a manner that the N- and C-termini of the protein are on the same inner side of the cell membrane. The transmembrane domains of all 4 transporters are composed of 12 α-helices and contain substrate-binding sites in the central region. However, these sites are different in size and structure and, as a result, possess different substrate specificities.

**Figure 1:**
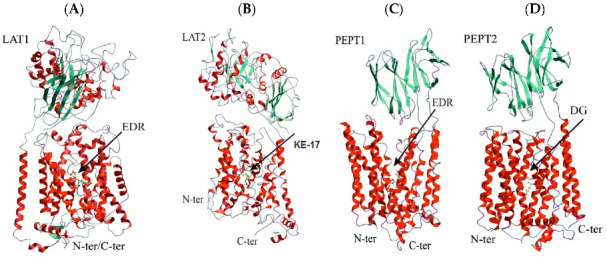
Spatial structures of the lowest-energy complexes of LAT1, LAT2, PEPT1, and PEPT2 transporters with USPs. **A**—complex of LAT1 and EDR peptide, **B**—complex of LAT2 and KE-17 peptoide, **C**—complex of PEPT1 and EDR peptide, **D**—complex of PEPT2 and DG peptide.

The docking results of the 26 biologically active USPs to the active sites of LAT1 and PEPT1 showed that the EDR peptide has the lowest value of the ICM-Score function (Figure 1A,C). This may be indicative of its ability to be transported into the cell via these transporters. These data are important for explaining the molecular mechanism of the neuroprotective effect of the EDR peptide. In vitro and in vivo studies revealed the efficacy of the EDR peptide in neurodegenerative diseases. In addition, the EDR peptide is administered orally in older age groups as a part of the complex therapy in cerebral pathology, and in athletes, to increase endurance [26,27]. The KE-17 peptoid (Figure 1B), a KE dipeptide with a modified peptide bond, possessing retinal protective properties, has the highest LAT2 affinity among the investigated USPs. This dipeptide is administered orally in the complex therapy of retinal degeneration. The ability of the KE-17 peptoid to stimulate the differentiation of the pigment epithelium, photoreceptors, and retinal neurons underlies its molecular mechanism of action [28]. The DG dipeptide is most likely to bind to the PEPT1 transporter (Figure 1D).

Thus, depending on the structure and conformation of the transporter’s active site and a USP, there are differences in the probability of USPs’ transport by various carriers of short peptides and amino acids.

### 3.2. Binding of USPs to the LAT1 Amino Acid Transporter

**Figure 2:**
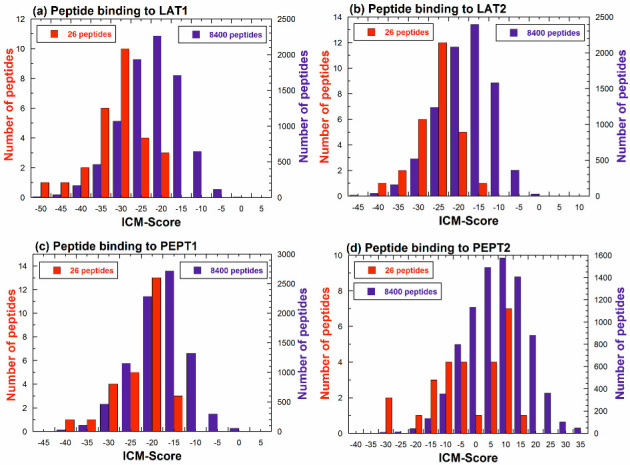
Comparative analysis of the binding characteristics of the 26 biologically active peptides and all possible di- and tri-peptides at the active sites of LAT1 (panel (**a**)), LAT2 (panel (**b**)), PEPT1 (panel (**c**)), and PEPT2 (panel (**d**)).

To analyze the probability of random binding of the 26 biologically active peptides, a large-scale docking of all possible di- and tri-peptides into the active sites of LAT1, LAT2, PEPT1, and PEPT2 transporters was carried out. The binding analysis of all possible di- and tri-peptides (8400 compounds in total) at the LAT1-binding site showed that all of the 26 biologically active USPs under investigation fall into the top 50% of the 8400 peptide ligands with the length of 2 and 3 amino acid residues in terms of their binding efficiency to LAT1 (Figure 2). The probability of a random coincidence in this case approximately equals (1/2)^26^ = 0.0000000149, which is an important argument in favor of the LAT1 involvement in the transport of the 26 USPs under investigation.

Table 1, Table 3, Table 5 and Table 7 demonstrate the docking results of the 26 USPs at the LAT1-, LAT2-, PEPT1-, and PEPT2-binding sites. To compare and evaluate the possible binding constants of USPs to these transporters, the docking data and experimentally measured binding constants for inhibitors of these transporters are listed in Table 2, Table 4, Table 6 and Table 8. As can be seen from the presented data, the ICM-Score values obtained during the docking of the 26 USPs match, and in many cases, exceed the binding efficiency characteristics for the inhibitors presented in Table 5, Table 6, Table 7 and Table 8. This indicates a higher binding quality of the 26 USPs to the transporters under study.

Figure 2 shows the distributions of ICM-Score values for complexes of the 26 biologically active USPs and all possible (8400 in total) di- and tri-peptides with LAT1, LAT2, PEPT1, and PEPT2 transporters. As can be seen from the presented data, the ICM-Score values of the 26 USPs are systematically shifted to more negative values compared to di- and tri-peptides for complexes with LAT1, LAT2, and PEPT1. Statistical analysis with the Shapiro-Wilk normal distribution test and the T-test showed that all the distributions presented in Figure 2 are within the normal range, and the mean values of the ICM-Score for the 26 biologically active USPs are significantly lower than the mean values of the ICM-Score for the 8400 di- and tri-peptides (*p*-value << 0.05).

As one can see from the Figure 2, among the 8400 tested di- and tri-peptides, there are many USPs whose ICM-Score is comparable or even exceeds these characteristics of the 26 biologically active peptides. The peptides with the highest potential binding characteristics are in the case of (a) LAT1: DDW (−49.8), NQQ (−48.2), QFR (−46.9), EWR (−46.2), EDY (−46.1), RPR (−45.9), RER (−45.8), EDF (−45.2), EVR (−45.1), and DDF (−45.1), (b) LAT2: SQT (−44.1), SHN (−42.8), DRN (−42.8), DFN (−42.2), RYN (−41.3), DGN (−41.1), QGE (−40.9), NEN (−40.4), EEN (−40.1), and DEN (−40.1), (c) PEPT1: QHR (−40.5), NPR (−39.5), ERD (−38.6), GGR (−38.5), PPR (−38.2), QGR (−37.8), FNR (−37.6), CER (−37.5 ), KQR (−37.5), and YHR (−37.4), and (d) PEPT2: EWC (−34.0), DFS (−31.6), DYT (−29.6), RD (−29.6), DWA (−28.6), DWP (−28.5), and EWR (−28.3). However, nothing is known of any biological activity of these and many other USPs having high values of the ICM-Score. In principle, there is nothing surprising in such a situation. The main function of the carriers of the LAT and PEPT families is the transport of a wide range of low molecular weight compounds and USPs for various purposes through cell membranes.

Interestingly, all the complexes of the 26 biologically active USPs, with the exception of complexes with the PEPT2 transporter, are located on the left—in the more negative part of the distribution of di- and tri-peptide ICM-Score values. The probability of such a random coincidence is very low, 1/2^26^ ~ 0.000000015, which means that the binding characteristics of the 26 biologically active peptides to the transporters under examination are better than for the set of all possible di- and tri-peptides on average. This observation is another indicator that the composition of the 26 biologically active peptides is not a random set of USPs, which may be due to transport of the biologically active peptides across the cell membrane via the considered transporters of the LAT and PEPT families. Indeed, if the 26 USPs under consideration had no relation to these transporters, then its distribution of ICM-Scores is expected to be close to the distribution of the 8400 di- and tri-peptides. Data shown in Figure 2 also show that some other di- and tri-peptides may also be substrates or inhibitors of these transporters.

**Figure 3:**
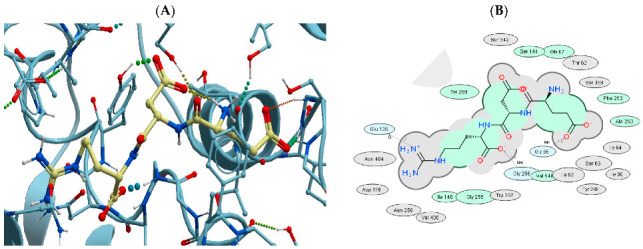
Structure ((**A**) three-dimensional, (**B**) two-dimensional) of the lowest-energy complex of the EDR peptide with the LAT1 transporter. The green shading represents the areas of hydrophobic interactions, and the gray shading represents the areas of Van der Waals contacts.

Figure 3 shows a map of the EDR peptide interactions at the binding site of the LAT1 amino acid transporter. The two-dimensional scheme demonstrates the interaction of the tripeptide and the amino acid residues of the LAT1 active site. The size of the LAT1 active site is sufficient for binding not only amino acids, but di- and tri-peptides as well. The mechanism of substrate transfer by LAT1 involves large-scale conformational rearrangements of the entire transporter structure with simultaneous closing of its external input channel and opening of the internal output channel. Therefore, it is quite possible that the presence in the LAT1 active site of di- and tri-peptides, which are larger than amino acids, may create steric hindrances for this substrate transfer mechanism. However, even in this case, biologically active peptides with low ICM-Score values presented in the table can serve as highly effective inhibitors of LAT1. As contrasted with dipeptides, tripeptides’ binding characteristics to the LAT1 transporter are much higher, and therefore tripeptides appear as preferred candidates for the anticancer drug development.

**Table 1:** Results of docking of the 26 biologically active USPs to the active site of the neutral amino acid transporter LAT1.

No.	Two-Dimensional Structure of the Peptide/Peptoid	Amino Acid Sequence or Peptide/Peptoid Name	Receptor in the PDB	ICM-Score *
1	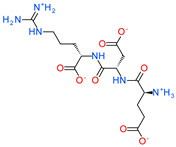	EDR	7DSK	−45.32
2	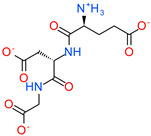	EDG	7DSK	−43.40
3	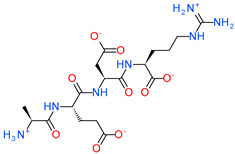	AEDR	7DSN	−35.35
4	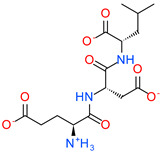	EDL	7DSK	−35.03
5	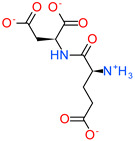	ED	6IRS	−34.55
6	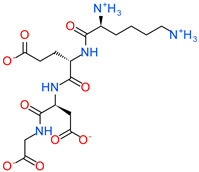	KEDG	6IRT	−34.05
7	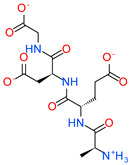	AEDG	6IRT	−32.93
8	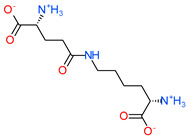	AB-17	7DSL	−32.76
9	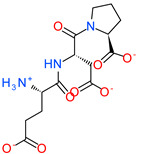	EDP	7DSK	−32.60
10	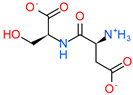	DS	6IRT	−30.49
11	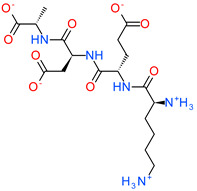	KEDA	6IRT	−28.83
12	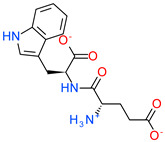	EW	7DSK	−28.72
13	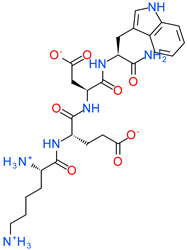	KEDW-NH2	6IRT	−28.40
14	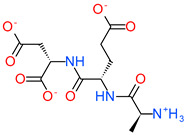	AED	6IRS	−28.14
15	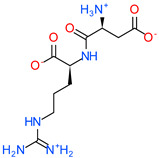	DR	6IRT	−28.01
16	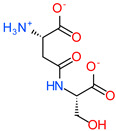	DS7	7DSL	−27.38
17	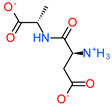	DA	6IRS	−27.25
18	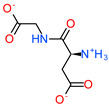	DG	6IRT	−26.49
19	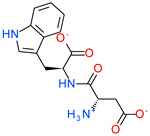	DW	7DSN	−25.18
20	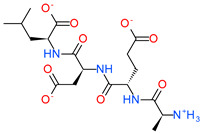	AEDL	6IRS	−25.11
21	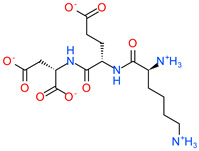	KED	6IRS	−24.79
22	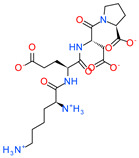	KEDP	6IRS	−22.79
23	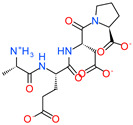	AEDP	7DSK	−20.95
24	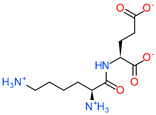	KE	7DSK	−19.02
25	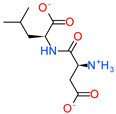	DL	7DSK	−16.70
26	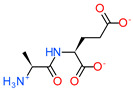	AE	7DSL	−16.59

* Preliminary results of USP docking at the LAT1 active site are presented in a recently published review of the literature on the structure and biological activity of the LAT and POT family transporters [4]. The data obtained in this work differ somewhat from preliminary calculations due to the use of several different conformations of the LAT1 active site, which is observed in various LAT1 structures available in PDB (PDB ID: 6IRS), as well as the use of new, more advanced techniques for docking mobile ligands with increased thoroughness.

**Table 2:** Inhibitors of the neutral amino acid transporter LAT1 with known IC_50_ values used for the l-Leu transport inhibition in *Pichia pastoris* cells and proteoliposomes.

No.	Two-Dimensional Structure of the Inhibitor	Name of the Inhibitor	Receptor in the PDB	ICM-Score	IC_50_ µM	Reference
1	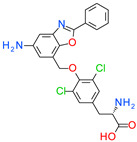	(2S)-2-amino-3-[4-[(5-amino-2-phenyl-1,3-benzox (JPH203)	7DSK	−31.50	0.35	[29]
2	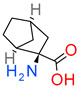	2-Amino-2-norbornanecarboxylic acid (BCH)	6IRS	−17.37	78.0	[29]
3	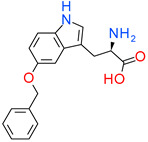	5-(benzyloxy)-tryptophan	7DSK	−25.25	1.48 *	[10]
4	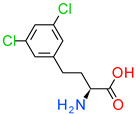	l-2-amino-4-(3,5-dichlorophenyl)-butanoic acid	7DSK	−19.84	0.62 *	[10]
5	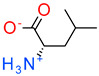	l-Leucine	7DSN	−23.96	9.0	[29]
6	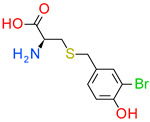	S-(3-bromo-4-methoxybenzyl)-l-cysteine	7DSK	−23.44	33.2	[30]
7	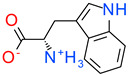	Tryptophan	7DSN	−23.17	20.2	[30]

* IC_50_ value is measured for the L-His transport inhibition in proteoliposomes. The correlation coefficient between the ICM-Score and IC_50_ values is R = ~0.64. If only L-Leu transport inhibition data are used, then R = ~0.93.

### 3.3. Binding of USPs to the LAT2 Amino Acid Transporter

Similar to LAT1, LAT2 also provides transport of neutral amino acids across the cell membrane. The transporters have high similarity in amino acid sequences and spatial structure. The available spatial structures of human LAT1 and LAT2 transporters exhibit similar inward-open conformations, indicating a common substrate recognition mechanism shared by LAT1 and LAT2 [29,30,31]. However, LAT2, which is mainly expressed in the kidneys and small intestine, has a wider range of substrates than LAT1. For example, in addition to large neutral amino acids such as Phe, Ile, Leu, and Trp, LAT2 can also transport small neutral amino acids such as Ala, Ser, and Thr [31]. Comparison of the substrate-binding pockets of the transporters LAT1 and LAT2 shows that the main differences are related to Ser96, Trp405, Val408, and Phe439 (LAT1) and the corresponding Thr86, Tyr396, Tyr399, and Tyr430 (LAT2), which may explain the observed differences in the substrate specificity of these transporters.

**Figure 4:**
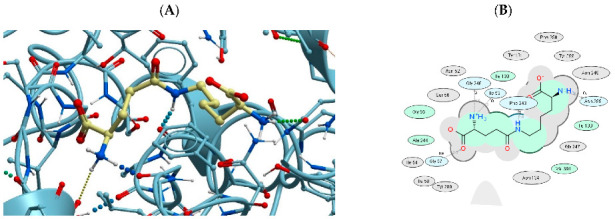
Structure ((**A**) three-dimensional, (**B**) two-dimensional) of the lowest-energy complex of the AB-17 peptoid with the LAT2 transporter. The green shading represents the areas of hydrophobic interactions, and the gray shading represents the areas of Van der Waals contacts.

The most efficient binding to LAT2 was found for the AB-17 peptoid, which has two negatively charged COO^−^ groups (Figure 4). Next, in terms of the probability of binding to LAT2 are the peptides: EDR (the best LAT1 ligand), ED, DS7, DS, DR, AEDR, EDG, and KEDG. These peptides have negatively charged Asp^−^ or Glu^−^ amino acid residues at the N-terminus and neutral or positively charged residues at the C-terminus of the peptide. The presence of the ED motif in the structure apparently improves the USPs’ binding characteristics to LAT2. The same sequence motif is present in the case of peptide binding by LAT1. Since these two transporters share a high level of structure similarity and a similar set of the best binders, one can conclude a high similarity of the structure of their binding sites, which suggests the presence of a large number of positively charged hydrogen bond donors for binding peptides containing the ED motif.

The same pattern is observed when peptides bind to LAT1, which confirms their structural and functional similarity. Moreover, these results indicate the correctness of the parameters chosen for the stochastic ligand docking procedures. As a result, no gaps in the low-energy conformations of the studied peptides at the binding sites of LAT1 and LAT2 were allowed, and the set of conformers used of the LAT1 and LAT2 transporters corresponded to the actual mobility of these proteins.

Figure 4 presents the AB-17 peptoid interaction map at the LAT2-binding site. The two-dimensional scheme reflects the interaction between the peptoid and amino acid residues of the LAT2 active site. Table 3 presents the results of docking of the 26 USPs at the LAT2 transporter-binding site. To compare and evaluate the probable binding constants of USPs to LAT2, docking data and experimentally measured binding constants of LAT2 substrates and inhibitors are presented in Table 4. The ICM-Score values obtained for the binding of the 26 USPs matched, and in many cases exceeded the binding efficiency parameters for all LAT2 substrates presented in Table 4.

**Table 3:** Results of docking of the 26 biologically active USPs to the active site of the LAT2 amino acid transporter.

No.	Amino Acid Sequence or Peptide/Peptoid Name	Receptor in the PDB	ICM-Score
1	AB-17	7B00	−39.60
2	EDR	7CMH	−32.04
3	ED	7B00	−31.35
4	DS7	7CMI	−29.29
5	DS	7CMI	−29.17
6	DR	7CMH	−27.98
7	AEDR	7CMI	−27.54
8	EDG	7B00	−26.82
9	KEDG	7CMI	−26.57
10	KE	7CMH	−23.94
11	DA	7B00	−23.73
12	AED	7CMH	−23.64
13	AEDG	7CMH	−23.62
14	AEDP	7CMI	−22.15
15	DW	7CMH	−22.08
16	KEDW-NH2	7B00	−21.78
17	KEDA	7CMH	−21.45
18	EW	7CMI	−20.12
19	KED	7CMH	−20.09
20	DG	7CMI	−20.08
21	DL	7CMH	−19.05
22	AEDL	7CMH	−18.88
23	EDP	7CMI	−18.75
24	EDL	7CMH	−17.37
25	AE	7CMH	−16.87
26	KEDP	7B00	−11.68

**Table 4:** Inhibitors of the LAT2 amino acid transporter with known IC_50_ values used for the l-Leu transport inhibition in *Pichia pastoris* cells [29].

No.	Two-Dimensional Structure of the Inhibitor	Inhibitor Name	Receptor in the the PDB	ICM-Score	IC_50_ µM
1	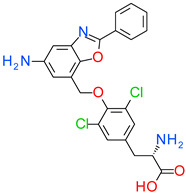	(2S)-2-amino-3-[4-[(5-amino-2-phenyl-1,3-benzox (JPH203)	7B00	−34.69	1.7
2	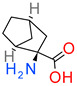	2-Amino-2-norbornanecarboxylic acid (BCH)	7B00	−15.15	184.0
3	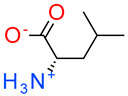	L-Leucine	7CMH	−27.78	39.0

The correlation coefficient between the ICM-Score and IC_50_ values is R = ~0.99.

### 3.4. Binding of USPs to the Di- and Tri-Peptide Transporter PEPT1

**Figure 5:**
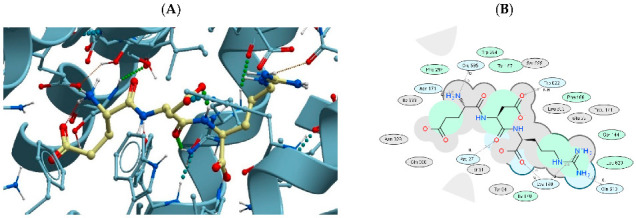
Structure ((**A**) three-dimensional, (**B**) two-dimensional) of the lowest-energy complex of the EDR tripeptide with the PEPT1 transporter. The green shading represents the areas of hydrophobic interactions, and the gray shading represents the areas of Van der Waals contacts.

The most efficient binding to the PEPT1 transporter is characteristic of the EDR peptide, which has two negatively charged amino acids at the N-terminus and a positively charged one at the C-terminus (Figure 5). To compare and evaluate the USPs’ binding constants to PEPT1, the docking data and experimentally measured binding constants of PEPT1 substrates and inhibitors are presented in Table 6. The ICM-Score values obtained for the binding of the 26 USPs match, and in many cases exceed the binding efficiency for the PEPT1 substrates presented in Table 6. Thus, the investigated USPs can be considered as promising substrates or inhibitors of the PEPT1 transporter. Interestingly, the ICM-Score values obtained for the interaction of USPs with LAT1 and LAT2 amino acid transporters were lower than the ICM-Score values of the USPs’ interaction with the di- and tri-peptide transporter PEPT1.

**Table 5:** Results of docking of the 26 biologically active USPs to the active site of the di- and tri-peptide transporter PEPT1.

No.	Amino Acid Sequence or Peptide/Peptoid Name	Receptor in the PDB	ICM-Score
1	EDR	7PMX	−37.59
2	KEDP	7PMX	−33.42
3	AEDR	7PN1	−27.50
4	DS	7PN1	−26.17
5	DR	7PMX	−25.74
6	AEDL	7PN1	−25.35
7	KEDG	7PN1	−22.72
8	DG	7PMX	−22.22
9	KEDW-NH2	7PN1	−20.57
10	EDL	7PN1	−20.48
11	AE	7PMX	−20.05
12	EW	7PN1	−19.00
13	AED	7PN1	−18.95
14	DA	7PN1	−18.26
15	EDG	7PN1	−18.04
16	AEDG	7PN1	−18.00
17	DS7	7PN1	−17.57
18	EDP	7PN1	−17.29
19	KE	7PN1	−17.02
20	KEDA	7PN1	−16.70
21	KED	7PN1	−16.42
22	ED	7PN1	−15.76
23	AB17	7PMW	−15.25
24	DL	7PMX	−13.88
25	AEDP	7PN1	−12.65
26	DW	7PMW	−10.66

**Table 6:** Inhibitors of the di- and tri-peptide transporter PEPT1 with known IC_50_ values [32].

No.	Two-Dimensional Structure of the Inhibitor	Inhibitor Name	Receptor in the PDB	ICM-Score	IC_50_ µM
1	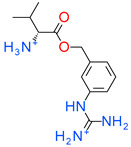	Val-[3-(hydroxymethyl)-phenyl]guanidine	7PN1	−27.74	650
2	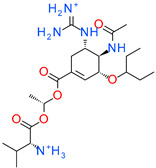	Guanidin-oseltamivir-carboxylate-L-Val-up	7PMX	−12.75	190 *
3	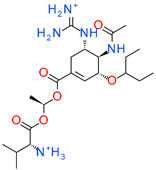	Guanidin-oseltamivir-carboxylate-L-Val-down	7PMX	−11.64	190 *
4	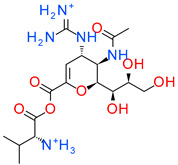	Val-Zanamivir	7PN1	−7.90	1190

* Weighted average IC_50_ value for two isomers of Guanidine-oseltamivir-carboxylate-l-Val.

### 3.5. Binding of USPs to the Di- and Tri-Peptide Transporter PEPT2

In humans, PEPT1 and PEPT2 transporters are similar in the structure and spectrum of functional activity [5,13,21,22]. The main difference is that the most pronounced PEPT1 expression is observed in the small intestine and kidneys. PEPT2 is predominantly localized in the kidneys. PEPT1 is a low-affinity transporter of di- and tri-peptides, while PEPT2 is a high-affinity transporter of USPs [26]. In this condition, it would be reasonable to expect that the results of docking of the 26 biologically active USPs into the active site of PEPT2 (Table 7) would be similar to those obtained for PEPT1 (Table 5), which turned out to be incorrect.

**Figure 6:**
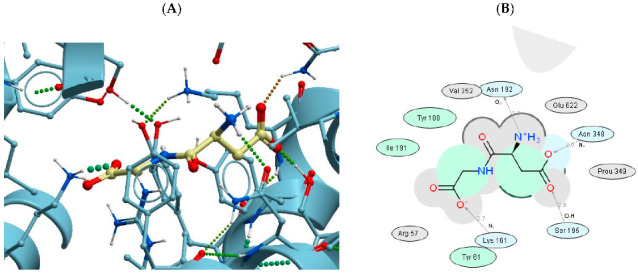
Structure ((**A**) three-dimensional, (**B**) two-dimensional) of the lowest-energy complex of the DG dipeptide with the PEPT2 transporter. The green shading represents the areas of hydrophobic interactions, and the gray shading represents the areas of Van der Waals contacts.

The size and amino acid composition of the PEPT2 transporter-binding site significantly differs from the PEPT1-binding site (Figure 6). Therefore, the set of highly effective PEPT2 ligands, which is dominated by dipeptides, differs from the ligands of the PEPT1 transporter. This contradicts the data available on the similarity of the functional activity of PEPT1 and PEPT2. It can therefore be concluded that the only spatial structure of human PEPT2 present in the PDB is not sufficiently representative of the conformational state in which the binding of the ligands of this transporter by di- and tri-peptides occurs. This is not surprising considering the high conformational mobility of all peptide transporters.

**Table 7:** Results of docking of the 26 biologically active USPs to the active site of the di- and tri-peptide transporter PEPT2 *.

No.	Amino Acid Sequence or Peptide/Peptoid Name	ICM-Score
1	DG	−27.16
2	DA	−25.92
3	ED	−18.22
4	DS	−13.39
5	DS7	−11.20
6	AE	−10.70
7	AED	−7.97
8	KEDP	−6.36
9	EDP	−5.40
10	EDG	−5.20
11	AB17	−4.19
12	DR	−3.80
13	EW	−0.78
14	AEDG	1.46
15	KE	6.40
16	KEDW-NH2	6.90
17	EDR	8.99
18	DL	9.17
19	DW	10.03
20	KED	11.14
21	KEDA	11.60
22	AEDL	12.13
23	KEDG	12.26
24	AEDR	13.15
25	EDL	13.48
26	AEDP	17.28

* Docking was carried out in the structure of the PEPT2 transporter (PDB ID: 7PMY).

**Table 8:** Inhibitors of the di- and tri-peptide transporter PEPT2 with known IC_50_ values [21].

No.	Two-Dimensional Structure of the Inhibitor	Inhibitor Name	Receptor in the PDB	ICM-Score	IC_50_ µM
1	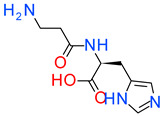	Carnosine	7PMY	−20.63	66
2	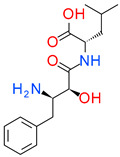	Bestatin	7PMY	−2.94	18
3	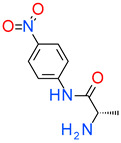	Alanine-4-nitroanilide	7PMY	−2.50	29

## 4. Discussion

The calculations performed indicate a high probability that the 26 biologically active USPs under investigation are transported through the cell membrane by LAT1, LAT2, and PEPT1 transporters. Alternatively, these peptides can act as transport inhibitors for other compounds transported by LAT1, LAT2, and PEPT1. In this case, peptides EDR, EDG, AEDR, EDL, KEDG, AED, EDP, and peptoid AB-17 are likely to be more potent inhibitors of the LAT1 amino acid transporter than the comparators. For them, the calculated value of the ICM-Score function was significantly lower than for known inhibitors of this transporter [10,29,30]. Apparently, due to the same reason, peptides DS, KEDA, EW, KEDW, EAD, AEDL, KE, and peptoid DS7 are more potent inhibitors of the LAT2 amino acid transporter [29].

It is known that an increase in the LAT1 synthesis and activity is a prognostic factor for an unfavorable development of malignant neoplasms. At the same time, selective inhibitors of LAT1 can contribute to the inhibition of the growth of carcinomas of various localization [33,34]. Antitumor activity of KE and AEDG peptides was shown in in vitro and in vivo studies [35,36,37,38]. It can be assumed that the antitumor effect of these short peptides is associated with their ability to inhibit LAT1 and LAT2 amino acid transporters.

The ICM-Score function value of the KEDP peptide (regulator of prostate function) is higher than that of known LAT2 inhibitors. Therefore, it can be assumed that this tetrapeptide will reversibly bind to LAT2 and be transported into the cell with its participation. In case this assumption is true, then smaller di- and tri-peptides are also likely to be transported via this carrier.

According to molecular modeling, ultrashort peptides AE, KE, ED, DA, DE, DG, DS, EW, DW, DL, KED, EDG, EDR, EDL, AED, EDP, KEDP, AEDG, AEDR, AEDL, AEDP, KEDW-NH_2_, KEDA, and KEDG are comparable to inhibitors of the PEPT1 transporter in terms of binding strength, since the calculated value of the ICM-Score function for them lies within the same range as for the known inhibitors [21,33]. At the same time, EDR and KEDP peptides are stronger PEPT1 inhibitors than the known compounds, since the ICM-Score function value for them is lower than for the reference substances.

Unfortunately, the data currently available on the PEPT2 transporter structure do not allow a reliable assessment of the binding efficiency of the 26 biologically active USPs at the active site of this transporter. It can be assumed that the small size of the active site of its only known conformation is only sufficient for the binding of peptide ligands no larger than two amino acid residues. However, this is most probably not the only possible PEPT2 conformation. This issue requires further detailed consideration based on more complete structural data on the conformational mobility of PEPT2.

## 5. Conclusions

Most of the 26 studied USPs of 2 and 3 amino acid residues in length bound to LAT1, LAT2, and PEPT1 transporters with more efficiency than known substrates or inhibitors of these transporters. The only spatial structure of the human PEPT2 present in the PDB was not sufficiently representative of the conformational state in which the binding of the ligands of this transporter by di- and tri-peptides occurred. Therefore, the results obtained on the binding of the 26 biologically active USPs can still be considered as preliminary only. Nevertheless, it was found that, according to the submitted calculations, DG and DA dipeptides can bind to PEPT2 more efficiently than known inhibitors of this transporter.

The most effective ligands of the LAT2 transporter were peptides EDR (the best binding ligand for LAT1 and PEPT1 transporters and the second most effective for LAT2), ED, DS, DR, AEDR, AEDL, EDG, KEDP, and KEDG, and peptoids DS7 and AB-17, with negatively charged Asp^−^ or Glu^−^ amino acid residues at the N-terminus and neutral or positively charged residues at the C-terminus. The presence of the ED motif in the structure apparently improved the binding characteristics of USPs to LAT1, LAT2, and PEPT1.

The antitumor effect of KE and AEDG peptides, previously revealed in in vitro and in vivo experiments, probably occurred due to their ability to inhibit LAT1, LAT2, and PEPT1 amino acid transporters.

The analysis of binding of all possible di- and tri-peptides (8400 compounds) at the active sites of LAT1, LAT2, and PEPT1 showed that the 26 biologically active USPs belong to 50% of the best peptide ligands, with lengths of 2 and 3 amino acid residues in terms of their binding efficiency to these transporters. This indicates the possibility of USPs’ transport through the cell membrane or inhibition of the active centers of the transporters themselves. Further experimental studies on the mechanisms of transport of the 26 biologically active USPs via the LAT and PEPT family transporters may be important for understanding the molecular background of peptide regulation of the physiological functions in the human body and creating new-generation drugs on this basis.

## Data Availability

Data is unavailable due to privacy.

## References

[1] Khavinson V.K., Popovich I.G., Linkova N.S., Mironova E.S., Ilina A.R. (2021). Peptide Regulation of Gene Expression: A Systematic Review. Molecules.

[2] Xiao Q., Dong X., Yang F., Zhou S., Xiang M., Lou L., Yao S.Q., Gao L. (2021). Engineered Cell-Penetrating Peptides for Mito-chondrion-Targeted Drug Delivery in Cancer Therapy. Chem. A Eur. J..

[3] Allolio C., Magarkar A., Jurkiewicz P., Baxová K., Javanainen M., Mason P.E., Šachl R., Cebecauer M., Hof M., Horinek D. (2018). Arginine-rich cell-penetrating peptides induce membrane multilamellarity and subsequently enter via formation of a fusion pore. Proc. Natl. Acad. Sci. USA.

[4] Khavinson V., Linkova N., Kozhevnikova E., Dyatlova A., Petukhov M. (2022). Transport of Biologically Active Ultrashort Peptides Using POT and LAT Carriers. Int. J. Mol. Sci..

[5] Campos-Bedolla P., Walter F.R., Veszelka S., Deli M.A. (2014). Role of the Blood–Brain Barrier in the Nutrition of the Central Nervous System. Arch. Med. Res..

[6] Li S., Whorton A.R. (2005). Identification of stereoselective transporters for S-nitroso-L-cysteine: Role of LAT1 and LAT2 in biological activity of S-nitrosothiols. J. Biol. Chem..

[7] Takahashi Y., Nishimura T., Higuchi K., Noguchi S., Tega Y., Kurosawa T., Deguchi Y., Tomi M. (2018). Transport of Pregabalin Via L-Type Amino Acid Transporter 1 (SLC7A5) in Human Brain Capillary Endothelial Cell Line. Pharm. Res..

[8] Goldenberg G.J., Lam H.Y., Begleiter A. (1979). Active carrier-mediated transport of melphalan by two separate amino acid transport systems in LPC-1 plasmacytoma cells in vitro. J. Biol. Chem..

[9] Widdows K.L., Panitchob N., Crocker I.P., Please C.P., Hanson M.A., Sibley C.P., Johnstone E.D., Sengers B.G., Lewis R.M., Glazier J.D. (2015). Integration of computational modeling with membrane transport studies reveals new insights into amino acid exchange transport mechanisms. FASEB J..

[10] Singh N., Ecker G.F. (2018). Insights into the Structure, Function, and Ligand Discovery of the Large Neutral Amino Acid Transporter 1, LAT1. Int. J. Mol. Sci..

[11] Meier C., Ristic Z., Klauser S., Verrey F. (2002). Activation of system L heterodimeric amino acid exchangers by intracellular substrates. EMBO J..

[12] Okano N., Hana K., Naruge D., Kawai K., Kobayashi T., Nagashima F., Endou H., Furuse J. (2020). Biomarker Analyses in Patients with Advanced Solid Tumors Treated with the LAT1 Inhibitor JPH203. In Vivo.

[13] Del Amo E.M., Urtti A., Yliperttula M. (2008). Pharmacokinetic role of L-type amino acid transporters LAT1 and LAT2. Eur. J. Pharm. Sci..

[14] Jeckelmann J.M., Lemmin T., Schlapschy M., Skerra A., Fotiadis D. (2022). Structure of the human heterodimeric transporter 4F2hc-LAT2 in complex with Anticalin, an alternative binding protein for applications in single-particle cryo-EM. Sci. Rep..

[15] Pineda M., Fernández E., Torrents D., Estévez R., López C., Camps M., Lloberas J., Zorzano A., Palacín M. (1999). Identification of a membrane protein, LAT-2, that co-expresses with 4F2 heavy chain, an L-type amino acid transport activity with broad specificity for small and large zwitterionic amino acids. J. Biol. Chem..

[16] Zevenbergen C., Meima M.E., Lima de Souza E.C., Peeters R.P., Kinne A., Krause G., Visser W.E., Visser T.J. (2015). Transport of Iodothyronines by Human L-Type Amino Acid Transporters. Endocrinology.

[17] Pinto V., Pinho M.J., Soares-da-Silva P. (2013). Renal amino acid transport systems and essential hypertension. FASEB J..

[18] Barollo S., Bertazza L., Watutantrige-Fernando S., Censi S., Cavedon E., Galuppini F., Pennelli G., Fassina A., Citton M., Rubin B. (2016). Overexpression of L-type amino acid transporter 1 (LAT1) and 2 (LAT2): Novel markers of neuroendocrine tumors. PLoS ONE.

[19] Chiu M., Sabino C., Taurino G., Bianchi M.G., Andreoli R., Giuliani N., Bussolati O. (2017). GPNA inhibits the sodium-independent transport system L for neutral amino acids. Amino Acids.

[20] Wang Q., Holst J. (2015). L-type amino acid transport and cancer: Targeting the mTORC1 pathway to inhibit neoplasia. Am. J. Cancer Res..

[21] Terada T., Sawada K., Irie M., Saito H., Hashimoto Y., Inui K.-I. (2000). Structural requirements for determining the substrate affinity of peptide transporters PEPT1 and PEPT2. Pflüg. Arch..

[22] Kamal M.A., Keep R.F., Smith D.E. (2008). Role and Relevance of PEPT2 in Drug Disposition, Dynamics, and Toxicity. Drug Metab. Pharmacokinet..

[23] Abagyan R., Totrov M. (1994). Biased probability Monte Carlo conformational searches and electrostatic calculations for peptides and proteins. J. Mol. Biol..

[24] Abagyan R., Orry A., Raush E., Totrov M. (2018). What constitutes a good docking score?. ICM-Pro User Guide v.3.8.

[25] Kolchina N., Khavinson V., Linkova N., Yakimov A., Baitin D., Afanasyeva A., Petukhov M. (2019). Systematic search for structural motifs of peptide binding to double-stranded DNA. Nucleic Acids Res..

[26] Ilina A., Khavinson V., Linkova N., Petukhov M. (2022). Neuroepigenetic Mechanisms of Action of Ultrashort Peptides in Alzheimer’s Disease. Int. J. Mol. Sci..

[27] Khavinson V.K., Linkova N.S., Umnov R.S. (2021). Peptide KED: Molecular-Genetic Aspects of Neurogenesis Regulation in Alzheimer’s Disease. Bull. Exp. Biol. Med..

[28] Khavinson V.K., Pronyaeva V.E., Linkova N.S., Trofimova S.V. (2013). Peptidergic Regulation of Differentiation of Embrionic Cells. Bull. Exp. Biol. Med..

[29] Kantipudi S., Fotiadis D. (2021). Yeast Cell-Based Transport Assay for the Functional Characterization of Human 4F2hc-LAT1 and-LAT2, and LAT1 and LAT2 Substrates and Inhibitors. Front. Mol. Biosci..

[30] Yan R., Zhao X., Lei J., Zhou Q. (2019). Structure of the human LAT1-4F2hc heteromeric amino acid transporter complex. Nature.

[31] Yan R., Zhou J., Li Y., Lei J., Zhou Q. (2020). Structural insight into the substrate recognition and transport mechanism of the human LAT2–4F2hc complex. Cell Discov..

[32] Saaby L., Nielsen C.U., Steffansen B., Larsen S.B., Brodin B. (2013). Current status of rational design of prodrugs targeting the intestinal di/tri-peptide transporter hPEPT1 (SLC15A1). J. Drug Deliv. Sci. Technol..

[33] Kaira K., Sunose Y., Ohshima Y., Ishioka N.S., Arakawa K., Ogawa T., Sunaga N., Shimizu K., Tominaga H., Oriuchi N. (2013). Clinical significance of L-type amino acid transporter 1 expression as a prognostic marker and potential of new targeting therapy in biliary tract cancer. BMC Cancer.

[34] Ohshima Y., Kaira K., Yamaguchi A., Oriuchi N., Tominaga H., Nagamori S., Kanai Y., Yokobori T., Miyazaki T., Asao T. (2016). Efficacy of system l amino acid transporter 1 inhibition as a therapeutic target in esophageal squamous cell carcinoma. Cancer Sci..

[35] Vinogradova I.A., Bukalev A.V., Zabezhinski M.A., Semenchenko A.V., Khavinson V.K., Anisimov V.N. (2008). Geroprotective effect of Ala-Glu-Asp-Gly peptide in male rats exposed to different illumination regimens. Bull. Exp. Biol. Med..

[36] Kossoy G., Zandbank J., Tendler E., Anisimov V., Khavinson V., Popovich I., Zabezhinski M., Zusman I., Ben-Hur H. (2003). Epitalon and colon carcinogenesis in rats: Proliferative activity and apoptosis in colon tumors and mucosa. Int. J. Mol. Med..

[37] Anisimov V.N., Khavinson V.K., Alimova I.N., Semchenko A.V., Yashin A.I. (2002). Epithalon Decelerates Aging and Suppresses Development of Breast Adenocarcinomas in Transgenic *HER-2/neu* Mice. Bull. Exp. Biol. Med..

[38] Anisimov V.N., Khavinson V.K., Mikhalski A.I., Yashin A.I. (2001). Effect of synthetic thymic and pineal peptides on biomarkers of ageing, survival and spontaneous tumour incidence in female CBA mice. Mech. Ageing Dev..

